# Association between serum advanced oxidation protein products and mortality risk in maintenance hemodialysis patients

**DOI:** 10.1186/s12967-021-02960-w

**Published:** 2021-06-30

**Authors:** Chun Zhou, Yuanyuan Zhang, Jianghua Chen, Changlin Mei, Fei Xiong, Wei Shi, Wei Zhou, Xusheng Liu, Shiren Sun, Jianwei Tian, Ziliang Ye, Qimeng Wu, Xianhui Qin, Jianping Jiang, Fan Fan Hou, CCSD (China Collaborative Study on Dialysis) group

**Affiliations:** 1 Division of Nephrology, National Clinical Research Center for Kidney Disease, State Key Laboratory of Organ Failure Research, Guangdong Provincial Key Laboratory of Renal Failure Research, Guangzhou Regenerative Medicine and Health Guangdong Laboratory, Guangdong Provincial Clinical Research Center for Kidney Disease, Nanfang Hospital, Southern Medical University, Guangdong Provincial Institute of Nephrology, Guangzhou, 510515 China; 2 Kidney Disease Center, College of Medicine, The First Affiliated Hospital, Zhejiang University, Hangzhou, China; 3 Division of Nephrology, Changzheng Hospital, Shanghai, China; 4 Department of Nephrology, Wuhan No. 1 Hospital, Tongji Medical College, Huazhong University of Science and Technology, Wuhan, China; 5 Department of Nephrology, Guangdong Provincial People’s Hospital, Guangdong Academy of Medical Sciences, Guangzhou, China; 6 Department of Nephrology, The 8th Medical Center of Chinese, PLA General Hospital, Beijing, China; 7 Departmentof Nephrology, Guangdong Provincial Hospital of Chinese Medicine (The Second Affiliated Hospital of Guangzhou University of Chinese Medicine), Guangzhou, China; 8 Department of Nephrology, Xijing Hospital, Xi’an, China

**Keywords:** Advanced oxidation protein products, All-cause mortality, Cardiovascular mortality, Hemodialysis patients

## Abstract

**Background:**

The association between serum advanced oxidation protein products (AOPP) and mortality risk remains equivocal. We aimed to assess the correlation of serum AOPP levels with the risk of all-cause mortality in hemodialysis (HD) patients.

**Methods:**

A total of 1394 maintenance HD patients with complete data on AOPP and related parameters were included from China Collaborative Study on Dialysis (CCSD), a multi-center, prospective cohort study. The primary outcome was all-cause mortality, the secondary outcome was cardiovascular disease (CVD) mortality.

**Results:**

During a median follow-up duration of 5.2 years (IQR, 2.1–5.4), all-cause mortality occurred in 492 (31.4%) participants. Overall, there was a reversed L-shaped association between serum AOPP and all-cause mortality in HD patients (*P* for nonlinearity = 0.04), with an inflection point at 87 µmol/L. Accordingly, there was no significant association between serum AOPP and all-cause mortality (per SD increment; HR, 0.94; 95%CI, 0.84, 1.05) in participants with AOPP < 87 µmol/L. However, there was a positive relationship of serum AOPP and all-cause mortality (per SD increment; HR, 1.24; 95%CI, 1.08, 1.42) in those with AOPP ≥ 87 µmol/L. Moreover, a similar trend was found for CVD mortality.

**Conclusions:**

Elevated serum AOPP levels were associated with higher risk of all-cause mortality in Chinese maintenance HD patients.

**Supplementary Information:**

The online version contains supplementary material available at 10.1186/s12967-021-02960-w.

## Introduction

Hemodialysis (HD) is a life-sustaining treatment for patients with end-stage kidney disease (ESKD). HD patients usually have a substantially increased risk of all-cause and cardiovascular disease (CVD) mortality [1, 2]. Since traditional risk factors could not account for all mortality risks in HD patients, it is of great clinical importance to identify more modifiable risk factors to reduce the mortality burden in HD patients.

Recently, a growing body of evidence suggested that HD is characterized by oxidative stress resulting from loss of antioxidants and accumulation of oxidative products during dialysis procedures [3–6]. Advanced oxidation protein products (AOPP), which result from the interaction between oxidants and plasma proteins, are considered reliable markers to estimate the degree of oxidant-mediated protein damage [7, 8]. Plasma levels of AOPP were higher in patients on hemodialysis, than those on peritoneal dialysis and general population [7]. However, few studies have reported the association between AOPP and the risk of mortality with inconsistent results and have not assessed AOPP continuously [9, 10]. Furthermore, the possible modifiers on the correlation of AOPP and mortality risk have not been evaluated in these studies.

Using data from China Collaborative Study on Dialysis (CCSD), a large scale, multi-center, prospective cohort study, we investigated the association of serum AOPP with all-cause, and CVD mortality, and examined the modifiers for the association in patients undergoing HD.

## Methods

### Participants and design

The baseline data of CCSD has been reported elsewhere [11–14]. In summary, CCSD is a multi-center study, performed in 9 large dialysis facilities (at least 200 HD patients in each facility) in 6 cities of China (Beijing, Shanghai, Guangzhou, Hangzhou, Wuhan, and Xi’an). Eligible participants were men and women aged ≥ 18 years, and with ESKD undergoing dialysis between January 1, 2005, and December 1, 2010. Excluded patients were those with uncompleted data or dialysis duration less than 3 months. The current cohort study enrolled 1,567 eligible HD participants from the CCSD, followed from July, 2010 to February, 2016 (Additional file 1: Figure S1). This study was approved by the local ethics committee in each center, and all participants provided written informed consent.

### Data collection, measurements, and follow-up

Baseline data of the present study were derived from the database of CCSD. All data were collected at enrollment on the basis of review of medical records by two experienced doctors and dialysis research nurses. The data, including demographic data, underlying renal diseases, medication records, dialysis modality, dialysis program, and cardiovascular morbidity, which was defined as the presence of clinically diagnosed ischemic heart disease, heart failure, and/or stroke after initiation of dialysis, were collected.

Blood pressure measurement was taken by sphygmomanometer before each of the three HD sessions, three times at 1 min intervals, all after 10 min of rest in a supine decubitus position. The mean of the three readings was calculated [15].

Participants were scheduled for follow-up every 1–3 months in each center. At each follow-up visit, possible endpoint events were documented by trained research staff and physicians.

### Hemodialysis regimens

Participants were dialyzed twice or thrice weekly with low-flux polysulphone or polyacrylamide dialyzer, either 1.5 or 1.7 m^2^ (Fresenius, Germany; Gambro, Sweden; Nipro, Japan; B. Braun, Germany; Langsheng, China). All treatments were of 4 h to 5 h duration with conventional glucose-free, bicarbonate-based dialysate containing 1.25–1.5 mM calcium, 2.0 mM potassium and 138 mM sodium. Dialysate flow was 500 mL/min.

### Laboratory assays

Baseline fasting venous serum specimens were collected before the hemodialysis sessions. Biochemical tests were performed by the clinical laboratories in individual local dialysis facilities, using automatic clinical analyzers following the same standard protocol.

Measurement of serum AOPP was performed in the central laboratory based on a spectrophotometric detection as previously described [11]. To avoid the confounding effect of endogenous compounds such as triglyceride on measurement, fasting serum samples were collected, centrifuged, stored in aliquots with delipidating procedures without repeat frozen and thaw [16–18]. For measurement, 10 mL of serum diluted 1:10 with phosphate-buffered saline (PBS), 200 mL of chloramine T (0–100 mol/L) for calibration and 200 mL of PBS as blank were applied on a microlitre plate. Ten microlitres of 1.16 M potassium iodide and 10 mL of acetic acid were added and absorbance at 340 nm was measured immediately. Concentration of AOPP is expressed in micromoles per litre (µmol/L) of chloramines-T equivalent. The coefficients of intra- and inter-assay variations were 1.95% and 3.7%, respectively.

### Study outcomes

All-cause mortality was the primary outcome, which included death due to any reason. The secondary outcome was CVD mortality, which included sudden cardiac death, stroke, myocardial infarction (MI), heart failure, and death due to other known vascular causes. Evidence for death included death certificates from hospitals or reports from investigator visits.

### Statistical analysis

Baseline characteristics are presented as the mean ± standard deviations (SDs) or median (interquartile range) for continuous variables and proportions for categorical variables, respectively. Differences in population characteristics according to categories (< 87 versus ≥ 87 µmol/L) of baseline AOPP were compared using *t* test, Wilcoxon rank sum test, or Chi-squared tests, accordingly.

There were missing values on hemoglobin (n = 30), white blood cell (n = 30), calcium (n = 36), phosphate (n = 44) at baseline. Multiple imputations were used to handle missing values at baseline in the outcome analyses. The association between serum AOPP and all-cause mortality and CVD mortality were estimated using Cox proportional hazard regression models, without and with adjustments for age, sex, body mass index (BMI), smoking, dialysis vintage, hemoglobin, phosphate, iron supplementation, use of phosphorus binder, study center, CVD status, hypertension status and diabetes status at baseline. The proportional hazards’ assumption was checked using statistical tests based on the scaled Schoenfeld residuals. We first conducted restricted cubic spline (RCS) Cox regression, with 4 knots (20th, 40th, 60th, 80th percentiles of AOPP), to test for linearity and explore the shape of serum AOPP with all-cause mortality and CVD mortality. We then used segmented regression that is using a separate line segment to fit each interval. Log-likelihood ratio test comparing one-line (non-segmented) model to segmented regression model was used to determine whether threshold exists. The inflection points that connecting the segments was based on the model gives maximum likelihood, and it was determined using two steps recursive method. The detailed information for the determination of thresholds was shown in the supplemental file. Additionally, possible effect modifiers on the association between serum AOPP and all-cause mortality were evaluated by stratified analyses and interaction testing, using likelihood ratio test.

Two-tailed *P* < 0.05 was considered statistically significant in all analyses. All statistical analyses were performed using R software, version 4.0.1 (http://www.R-project.org/, accessed June 6, 2020).

## Results

### Characteristics of participants

As shown in the flow chart (Additional file 1: Figure S1), 1,835 participants were in the follow-up study. Of those, a total of 1,567 HD patients with complete data on AOPP at baseline, were included in the final analysis. During the follow up, patients with kidney transplant (N = 183), transfer to peritoneal dialysis (N = 17), or lost to follow-up (N = 1), were censored.

Baseline demographic, clinical and laboratory characteristics of the included patients by categories of baseline serum AOPP were illustrated in Table 1. The mean age of the patients was 55.9 ± 15.3 years old, 57.2% of the patients were males, and the median dialysis duration was 30.8 months with interquartile range of 13.7 to 63.5 months. The mean serum AOPP level was 78 ± 22 µmol/L. Patients with higher AOPP levels were more likely to be older, had longer duration of dialysis, higher BMI, C-reactive protein, hemoglobin, white blood cells, phosphate, total cholesterol, and TG levels. In addition, patients with higher AOPP levels had a higher frequency of using glucose-lowering drugs, phosphorus binder and a higher prevalence of diabetes at baseline.

**Table 1:** Baseline characteristics of the participants by serum AOPP categories

Variables	Baseline serum AOPP, µmol/L		*P*-value
	< 87	≥ 87	
N	1126	441	
Male, no. (%)	649 (57.6)	247 (56.0)	0.558
Age, year	55.0 ± 15.6	58.4 ± 14.2	< 0.001
Dialysis vintage, month	28.9 (11.8–60.5)	35.1 (18.6–67.9)	< 0.001
BMI, kg/m^2^	21.3 ± 3.3	22.2 ± 3.7	< 0.001
MAP, mmHg	103.1 ± 14.0	101.6 ± 14.3	0.055
Smoking, no. (%)	134 (11.9)	66 (15.0)	0.102
CVD, no. (%)	562 (49.9)	241 (54.6)	0.092
Diabetes, no. (%)	557 (49.5)	262 (59.4)	< 0.001
Hypertension, no. (%)	1028 (91.3)	410 (93.0)	0.278
Laboratory results			
C-reactive protein, mg/L	3.4 (1.8–10.3)	4.7 (2.9–10.3)	0.011
Albumin, g/L	39.7 ± 6.3	39.6 ± 6.8	0.886
Hemoglobin, g/L	102.4 ± 20.5	105.4 ± 18.7	0.008
White blood cells, 10^9^/L	5.9 ± 1.9	6.2 ± 2.0	0.009
Calcium, mmol/L	2.2 ± 0.3	2.3 ± 0.3	0.055
Phosphate, mmol/L	1.9 ± 0.7	2.1 ± 0.7	< 0.001
iPTH, pg/mL	258 (131.2–500.3)	280.7 (138.5–608.9)	0.147
Total cholesterol, mmol/L	4.0 ± 1.0	4.4 ± 1.2	< 0.001
TG, mmol/L	1.1 (0.8–1.6)	1.8 (1.1–2.7)	< 0.001
AOPP, μmol/L	68 ± 11	105 ± 19	< 0.001
Medication use, no. (%)			
ACEI/ARB	564 (50.1)	214 (48.5)	0.578
Glucose-lowering drugs	135 (12.0)	85 (19.3)	< 0.001
Lipid-lowering drugs	98 (8.7)	42 (9.5)	0.609
Antiplatelet drugs	146 (13.0)	51 (11.6)	0.452
Iron supplement	598 (53.1)	219 (49.7)	0.219
Phosphorus binder	439 (39.0)	205 (46.5)	0.007

Continuous variables are presented as Mean ± SDs or median (interquartile range), categorical variables are presented as no. (%)

*BMI* body mass index, *MAP* mean arterial pressure, *CVD* cardiovascular disease, *iPTH* parathyroid hormone, *TG* triglyceride, *AOPP* advanced oxidation protein products

Furthermore, in the multiple regression models, smoking status, dialysis vintage, phosphate, total cholesterol, and TG were significantly associated with serum AOPP (Additional file 1: Table S1).

### Association between AOPP and study outcomes

During a median follow-up duration of 5.2 years (IQR, 2.1–5.4), all-cause or CVD mortality occurred in 492 (31.4%) and 340 (21.7%) participants, respectively.

**Fig. 1:**
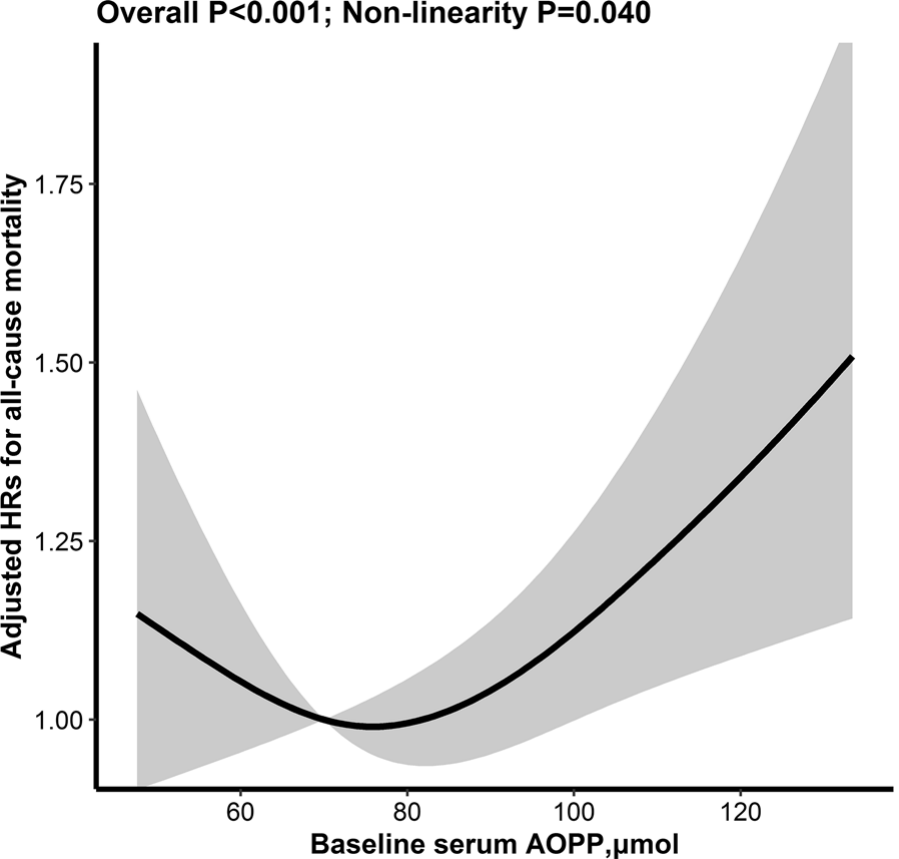
Restricted cubic spline for the association between baseline serum AOPP and all-cause mortality ^*^. ^*^Adjusted forage, sex, BMI, smoking, dialysis vintage, hemoglobin, phosphate, iron supplement, use of phosphorus binder, study center, CVD status, hypertension status and diabetes status at baseline. (dashed line represents the reference line of HR = 1.0, the black line represents the fitting curve of HRs for the association, the gray area represents 95% confidence intervals.)

Serum AOPP levels were significantly higher in the mortality group compared with the survival group (mean ± SDs, 81 ± 25 *vs.* 77 ± 20 µmol/L, *P* < 0.001). Overall, there was a reversed L-shaped association between serum AOPP and all-cause mortality in HD patients (*P* for nonlinearity = 0.04), with an inflection point at 87 µmol/L. (Fig. 1). There was no significant association between serum AOPP and all-cause mortality (per SD increment; HR, 0.94; 95%CI, 0.84, 1.05) in participants with AOPP < 87 µmol/L. However, there was a positive relationship of serum AOPP and all-cause mortality (per SD increment; HR, 1.24; 95%CI, 1.08, 1.42) in those with AOPP ≥ 87 µmol/L (Table 2). As expected, compared with those with AOPP < 87 µmol/L, a significantly higher risk of all-cause mortality was found in participants with AOPP ≥ 87 µmol/L (HR, 1.23; 95%CI: 1.02, 1.48; Additional file 1: Figure S3A). In the Cox proportional hazard regression models, no clear evidence was found against the proportional hazards’ assumption in the model (All *P* values > 0.05). Moreover, a similar trend was found with further adjustments for total cholesterol, TG, albumin, PTH, KT/V, and the use of EPO at baseline (Additional file 1: Table S2). Of note, the association between AOPP and all-cause mortality remained significant after Bonferroni multiple test correction for two tests (adjusted *P* = 0.025).

**Table 2:** Threshold effect analyses of serum AOPP (per SD increment) on all-cause mortality and CVD mortality using two-piecewise regression models

AOPP, µmol/L	Case	Incident rate (%)	Unadjusted model		Adjusted model*	
			HR (95% CI)	*P* value	HR (95% CI)	*P* value
All-cause mortality						
< 87	332	29.5	0.97 (0.87,1.08)	0.561	0.94 (0.84,1.05)	0.295
≥ 87	160	36.3	1.25 (1.10,1.43)	< 0.001	1.24 (1.08,1.42)	0.002^a^
CVD mortality						
< 88	242	21.0	0.95 (0.84,1.08)	0.421	0.89 (0.78,1.01)	0.068
≥ 88	98	23.7	1.19 (1.00,1.42)	0.046	1.26 (1.05,1.52)	0.015

*Adjusted for age, sex, BMI, smoking, dialysis vintage, hemoglobin, phosphate, iron supplement, use of phosphorus binder, study centers, CVD status, hypertension status and diabetes status at baseline

^a^For the primary outcome (all-cause mortality), since there are two times assessments, we used the Bonferroni method and accepted *P* < 0.025 as significant

A similar trend was found for the CVD mortality, with an inflection point at 88 µmol/L (Table 2, Additional file 1: Figures S2, S3B).

### Stratified analyses by potential effect modifiers

**Fig. 2:**
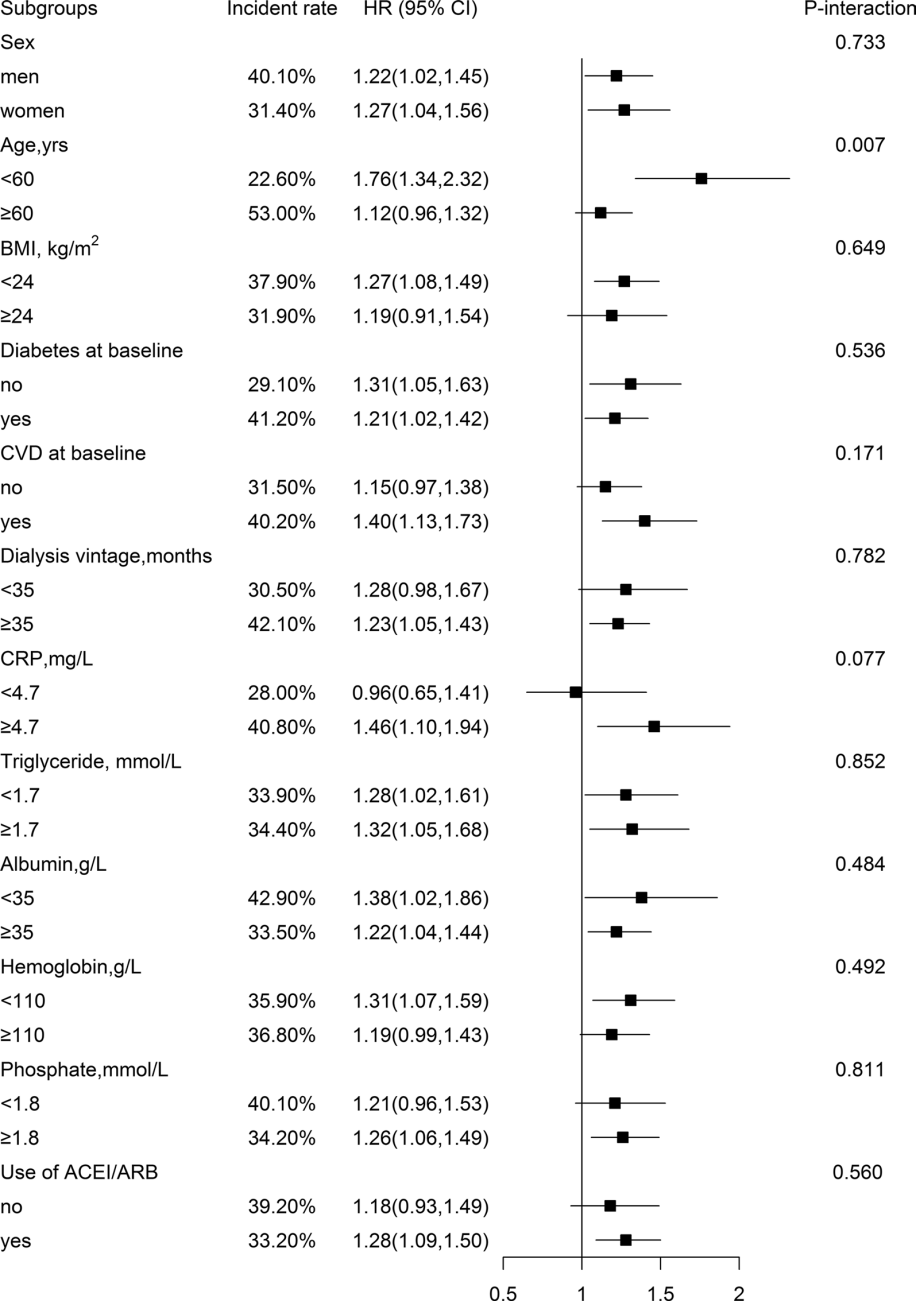
The association of baseline serum AOPP (per SD increment) with the risk of all-cause mortality among participants with AOPP ≥ 87 μmol/L in various subgroups *. *If not stratiied, adjusted for age, sex, BMI, smoking, dialysis vintage, hemoglobin, phosphate, iron supplement, use of phosphorus binder, study center, CVD status, hypertension status and diabetes status at baseline. Diabetes was deined as a fasting glucose ≥ 7.0 mmol/L or using glucose-lowering drugs or having history of diabetes

Among participants with AOPP ≥ 87 µmol/L, we further performed stratified analyses to assess the association between baseline AOPP levels (per SD increment) and risk of all-cause mortality in various subgroups (Fig. 2). A stronger association between AOPP levels and all-cause mortality was found in HD patients with younger age (< 60 years, adjusted HR, 1.76; 95%CI: 1.34, 2.32 *vs.* ≥ 60 years, adjusted HR, 1.12; 95%CI: 0.96, 1.32; *P*-interaction = 0.007).

None of other factors significantly modified the association between AOPP levels and risk of all-cause mortality in HD patients (all *P*-interactions > 0.05) (Fig. 2).

## Discussion

The present study first demonstrated that elevated baseline AOPP levels was significantly associated with higher risk of all-cause mortality in Chinese maintenance hemodialysis (MHD) patients. Moreover, a stronger association between AOPP and all-cause mortality was found in non-diabetic HD patients.

Few previous studies have been conducted to investigate the relation of AOPP with mortality, and the reported results have been inconsistent. One reported a positive link between AOPP levels and mortality risk in 540 non-HD, Brazilian elders [9]. However, another conducted in 112 HD patients, found that AOPP had no significant impact on survival in relatively young Brazilian HD patients [10]. Of note, due to the low sample size of the previous studies, the possible effect modifies for the AOPP-mortality association had not been fully examined in previous studies [9, 10]. Moreover, these two studies only assessed the association between AOPP and mortality by using AOPP as dichotomous variables (median, or ≤ 60 versus > 60 µmol/L), and did not allow for the possibility of non-linear association between AOPP and mortality. These results indicated that the association between AOPP and mortality risk remains inconclusive.

Our study provided an opportunity to evaluate the dose–response association between AOPP levels and the risk of mortality in HD patients with by far the largest sample size in any similar studies, and included a comprehensive adjustment and stratified analysis for a series of important confounders. Our study provided some new insights. First, there was a reversed L-shaped association serum AOPP and all-cause mortality in HD patients, with an inflection point at 87 µmol/L. That is to say that there was a threshold of serum AOPP level at about 87 µmol/L, above which the risk of all-cause mortality increased. In fact, some biological plausibility of the positive association between AOPP and mortality has been reported. MHD patients were reported to have imbalances between pro-inflammatory cytokines and their inhibitors, and between oxidants and anti-oxidants defense, resulting in a state of overwhelmed chronic oxidative stress [19–24]. Excess reactive oxygen species could induce damage to proteins, indicated by increased AOPP levels. Damaged proteins, could change enzyme levels and susceptibility to proteolytic, result in less active intracellular status, thereby contribute to structural and functional detriment of cells [25–28]. Moreover, oxidant-mediated protein damage may injure the anti-oxidant activity of albumin, and lead to the oxidative burst and synthesis of pro-inflammatory and inflammatory cytokines in human neutrophils and monocytes, then increase the accumulation of oxidants and inflammation [29]. Damaged cells along with oxidants and inflammation will accelerate atherosclerosis [30–32], which is closely related to coronary artery diseases [33], metabolic syndrome [34], and cancers [35–37], all might account for accumulating risk of mortality. However, more studies are warranted to confirm our findings and to further investigate the underlying mechanisms involved in the association between AOPP and mortality.

Second, among participants with AOPP ≥ 87 µmol/L, the stronger association of AOPP with all-cause mortality was found in younger MHD patients. It had been reported that aging was associated with an increase of cellular senescence and reactive oxygen species, which leads to oxidation, inflammation, and cell membrane [38]. A recent study further suggested that aging is a product of oxidative damage to mitochondrial DNA [39]. As expected, in our current study, older patients had significantly increased mortality risk. As such, we speculated that the high level of oxidative stress in older patients may possibly attenuate the positive association between higher AOPP and the risk of mortality. However, further studies are needed to verify this hypothesis and further investigate the underlying mechanisms.

Several shortcomings of the present study are needed to be considered. First, though a broad series of covariates were adjusted in the regression model, unmeasured or unknown residual confounding have not been fully considered. Second, the serum AOPP was only assessed once at baseline, thus the variability of AOPP during follow-up has not been taken into account. Third, we have not available data on antioxidant and antioxidant enzymes concentration. Therefore, we could not examine the association between antioxidant levels and AOPP, and evaluate whether theses variables may affect the relationship of AOPP with mortality risk. Fourth, the present study was conducted in Chinese hemodialysis patients, generalizability of the results to other ethnic or countries is still in need of consideration. As such, our findings should be further confirmed in more studies.

In conclusion, our study suggested that elevated serum AOPP levels were associated with higher risk of all-cause mortality in MHD patients. Our findings, if further confirmed, are highly relevant to clinical practice, in terms of early detection of the mortality risk in MHD patients.

## Supplementary Information

**Additional file 1.** Additional figures and tables.

## Data Availability

Not applicable.

## Abbreviations

AOPP: Advanced oxidation protein products
HD: Hemodialysis
MHD: Maintenance hemodialysis
CCSD: China Collaborative Study on Dialysis
CVD: Cardiovascular disease
ESKD: End-stage kidney disease
PBS: Phosphate-buffered saline
MI: Myocardial infarction
SDs: Standard deviations
BMI: Body mass index
HDL-C: High-density lipoprotein cholesterol
MBP: Mean blood pressure
CRP: C-reactive protein
iPTH: Parathyroid hormone
TG: Triglyceride
