# Supplementary material for: Association between serum advanced oxidation protein products and mortality risk in maintenance hemodialysis patients
Source: J Transl Med. 2021 Jun 30;19:284. doi: 10.1186/s12967-021-02960-w (PMC8247246; doi:10.1186/s12967-021-02960-w)
Supplement: Supplementary file 1 — Additional file 1. Additional figures and tables. [file 12967_2021_2960_MOESM1_ESM.doc]

1835 HD patients enrolled

in CCSD

Missing baseline serum AOPP : N=268.

1567 HD patients

for the final analysis

492 (31.4%) died during median of follow-up 5.2 years

**Supplementary Figure 1. Flow chart of the study participants**

**
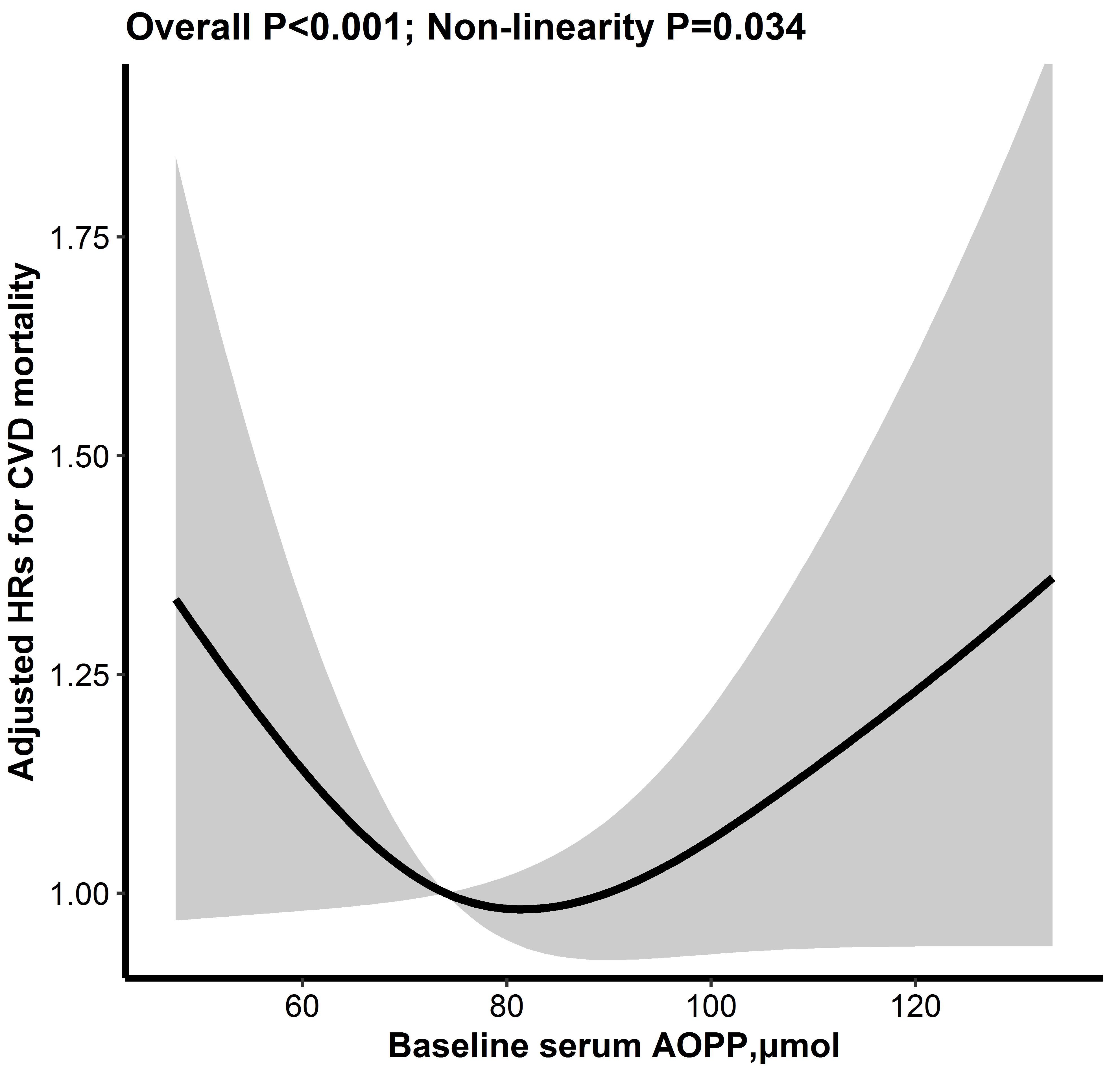
**

**Supplementary Figure 2. Restricted cubic spline for the association between baseline serum AOPP and CVD mortality ^*^**

^*^ Adjusted forage, sex, BMI, smoking, dialysis vintage, hemoglobin, phosphate, iron supplement, use of phosphorus binder, study center, CVD status, hypertension status and diabetes status at baseline.

**
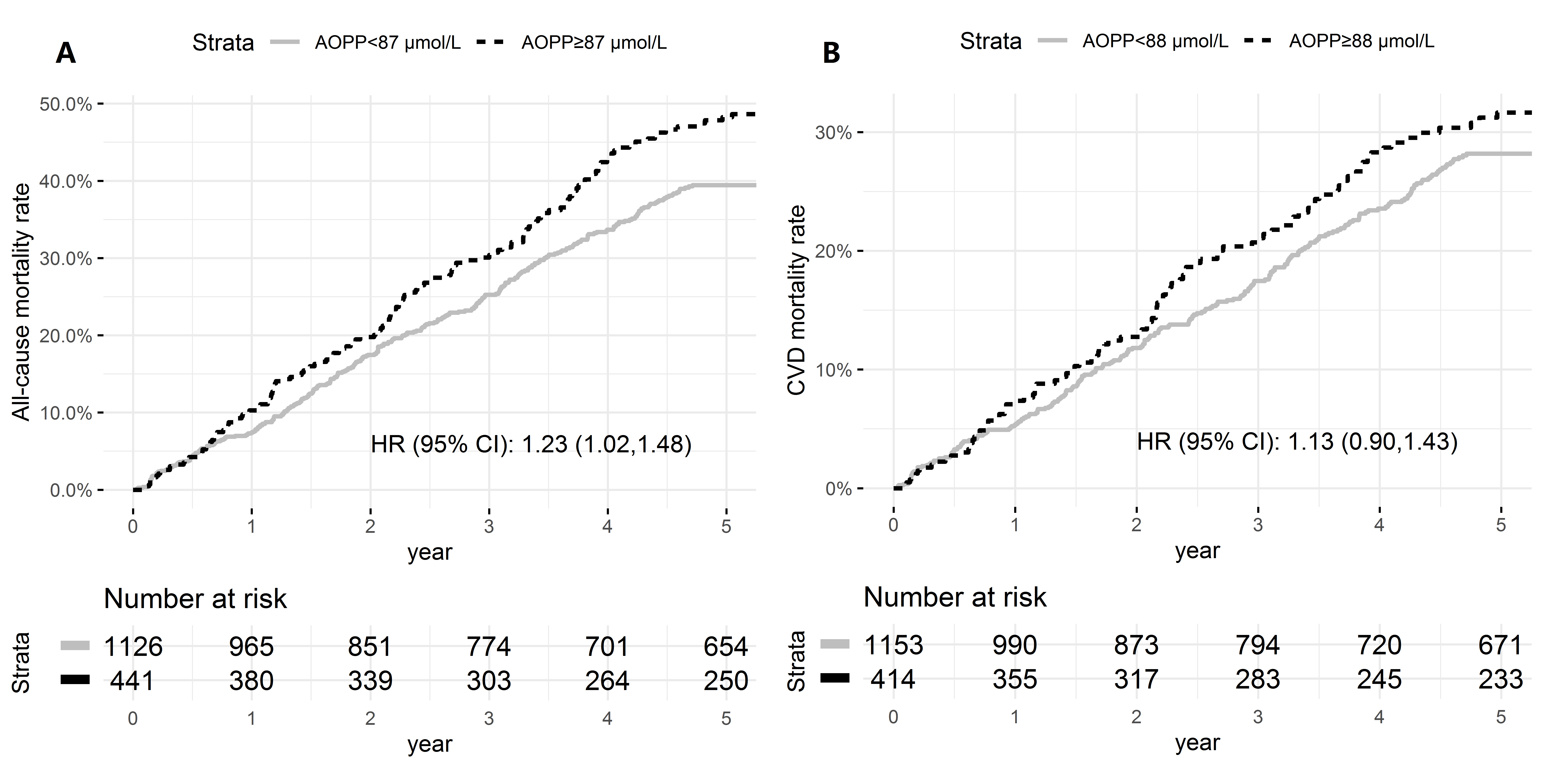
**

**Supplementary Figure 3. Kaplan-Meier survival curves for all-cause mortality (A) and CVD mortality (B).**

**Supplementary file:**

Detailed information for determination of the inflection point:

We apply segmented regression (also known as piece-wise regression) that is using a separate line segment to fit each interval. Log-likelihood ratio test comparing non-segmented model to segmented regression model was used to determine whether threshold exists. The inflection point that connecting the segments was based on the model giving maximum likelihood, and it was determined using two steps recursive method.

The step 1 is to narrow down the inflection point to a 10-percentile range of the independent variable. From 5% to 95% incremented by 5%, we test 19 segmented regression models using these 19 percentile points of independent variable as the inflection point respectively to find out which percentile points gives the model with highest likelihood. The precise inflection point was narrowed down to +/- 4% percentile of the percentile points which gives highest likelihood among the 19 models, called Kmin and Kmax respectively. The step 2 is to determine precise inflection point between Kmin and Kmax using the recursive method. The specific method is to first run 4 models with inflection point equals Q1 (25% percentile), Q2 (50% percentile) and Q3 (75% percentile) within the range of Kmin and Kmax respectively to find out which quartile point gives the model with highest likelihood among the three models. Then we narrow down the Kmin and Kmax to the range of +/- 25% of the corresponding quartile point. By doing so, we narrow down the range of Kmin and Kmax 50% recursively each time until the specific value of the independent variable was identified, that if used as inflection point will give the segmented regression model highest likelihood.

**Supplementary Table 1. Multi-variable linear regression for AOPP**

| Variables | β(95% CI) | *P*-value |
| --- | --- | --- |
| Age, year | 0.07 (-0.01,0.16) | 0.085 |
| Male, no. (%) | 0.03 (-2.48,2.55) | 0.979 |
| Dialysis vintage, month | 0.05 (0.02,0.08) | < 0.001 |
| BMI, kg/m^2^ | 0.03 (-0.32,0.38) | 0.869 |
| MAP, mmHg | -0.02 (-0.11,0.07) | 0.714 |
| Smoking, no. (%) | 5.60 (2.25,8.94) | 0.001 |
| CVD, no. (%) | 1.74 (-0.59,4.08) | 0.143 |
| Diabetes, no. (%) | 2.2 (-0.38,4.79) | 0.094 |
| Hypertension, no. (%) | 1.58 (-3.14,6.29) | 0.512 |
| **Laboratory results** |  |  |
| Albumin, g/L | 0.04 (-0.15,0.23) | 0.670 |
| Hemoglobin, g/L | 0.04 (-0.02,0.10) | 0.242 |
| White blood cells, 10^9^/L | 0.33 (-0.27,0.94) | 0.283 |
| Calcium, mmol/L | 3.14 (-1.33,7.60) | 0.168 |
| Phosphate, mmol/L | 2.89 (1.15,4.63) | 0.001 |
| iPTH, pg/mL | 0.00 (0.00,.0.00) | 0.848 |
| Total cholesterol, mmol/L | 1.44 (0.26,2.62) | 0.017 |
| TG, mmol/L | 5.83 (4.72,6.94) | < 0.001 |
| **Medication use, no. (%)** |  |  |
| ACEI/ARB | 0.09 (-2.29,2.47) | 0.939 |
| Glucose-lowering drugs | 1.06 (-2.53,4.65) | 0.564 |
| Lipid-lowering drugs | -3.89 (-8.18,0.41) | 0.076 |
| Antiplatelet drugs | -1.52 (-4.88,1.84) | 0.374 |
| Iron supplement | -0.91 (-3.24,1.43) | 0.446 |
| Phosphorus binder | 2.23 (-0.08,4.55) | 0.059 |

**Supplementary Table 2. Threshold effect analyses of serum AOPP (per SD increment) on all-cause mortality using two-piecewise regression models with further adjustment of total cholesterol, TG, serum albumin, serum PTH, Kt/V, and the use of EPO at baseline**

| AOPP, µmol/L | Unadjusted model | | Adjusted model^*^ | | |
| --- | --- | --- | --- | --- | --- |
|  | HR (95% CI) | *P* value | HR (95% CI) | *P* value | |
| **All-cause mortality** |  |  |  |  |  |
| <87 | 0.97 (0.87,1.08) | 0.561 | 0.99 (0.82,1.19) | 0.911 | |
| ≥87 | 1.25 (1.10,1.43) | < 0.001 | 1.32 (1.08,1.61) | 0.007 | |

**^*^** Adjusted for age, sex, BMI, smoking, dialysis vintage, hemoglobin, phosphate, iron supplement, use of phosphorus binder, study center, CVD status, hypertension status and diabetes status, total cholesterol, TG, serum albumin, serum PTH, Kt/V, and the use of EPO at baseline**.**
